# The Microbiota and It’s Correlation With Metabolites in the Gut of Mice With Nonalcoholic Fatty Liver Disease

**DOI:** 10.3389/fcimb.2022.870785

**Published:** 2022-05-27

**Authors:** Congwei Gu, Zihan Zhou, Zehui Yu, Manli He, Lvqin He, Zhengzhong Luo, Wudian Xiao, Qian Yang, Fangfang Zhao, Weiyao Li, Liuhong Shen, Jianhong Han, Suizhong Cao, Zhicai Zuo, Junliang Deng, Qigui Yan, Zhihua Ren, Mingde Zhao, Shumin Yu

**Affiliations:** ^1^ College of Veterinary Medicine, Sichuan Agricultural University, Chengdu, China; ^2^ Laboratory Animal Centre, Southwest Medical University, Luzhou, China

**Keywords:** nonalcoholic fatty liver disease (NAFLD), mice, 16SrDNA, metabonomics, MIMOSA2

## Abstract

In recent years, nonalcoholic fatty liver disease (NAFLD) has become the most common liver disease in the world. As an important model animal, the characteristics of gut microbiota alteration in mice with NAFLD have been studied but the changes in metabolite abundance in NAFLD mice and how the gut microbiota affects these intestinal metabolites remain unclear. In this experiment, a mouse model for NAFLD was established by a high-fat diet. The use of 16S rDNA technology showed that while there were no significant changes in the alpha diversity in the cecum of NAFLD mice, the beta diversity changed significantly. The abundance of *Blautia*, *Unidentified-Lachnospiraceae*, *Romboutsia*, *Faecalibaculum*, and *Ileibacterium* increased significantly in NAFLD mice, while *Allobaculum* and *Enterorhabdus* decreased significantly. Amino acids, lipids, bile acids and nucleotide metabolites were among the 167 significantly different metabolites selected. The metabolic pathways of amino acids, SFAs, and bile acids were significantly enhanced, while the metabolic pathways of PUFAs, vitamins, and nucleotides were significantly inhibited. Through correlation and MIMOSA2 analysis, it is suggested that gut microbiota does not affect the changes of lipids and bile acids but can reduce thiamine, pyridoxine, and promote L-phenylalanine and tyramine production. The findings of this study will help us to better understand the relationship between gut microbiota and metabolites in NAFLD.

## Introduction

Recently, nonalcoholic fatty liver disease (NAFLD) has become one of the most common liver diseases in the world. NAFLD is also one of the main causes of liver transplantation in the United States (Gadiparthi et al., 2020) and has replaced Viral Hepatitis B as the most common chronic liver disease in China (Xiao et al., 2019). The implication of the gut microbiota in the regulation of host metabolic balance has been demonstrated in the last decade. Many studies conducted in both animal models and humans revealed a significant role of the gut microbiota in the pathogenesis of metabolic disorders, strongly influenced by diet and lifestyle modifications. The gut microbiota recently emerged as a pivotal transducer of environmental influences (dietary components and drug treatments) to exert protective or detrimental effects on several host tissues and systems, including regulation of intermediary metabolism, liver function, and cardiovascular disorders, either directly *via* translocation or indirectly through microbial metabolism or their function in metabolic disorders (Bäckhed et al., 2004).

The overall composition of the gut microbiota is determined by a number of factors including host genetics, environment, and hygiene (Repass et al., 2016). The composition of the gut microbiome is influenced by environmental factors more so than by host genetics, where diet represents the predominate environmental factor influencing the makeup of the intestinal microbiome community (Pilla et al., 2020).Diets can directly interact with microorganisms to promote or inhibit their growth, and the capability to extract energy from specific dietary constituents bestows a direct competitive advantage to selected members of the gut microbial community, rendering them more capable of proliferating at the expense of less-adept members. Diets not only affects the absolute and relative abundance of gut bacteria but also their growth kinetics (Korem et al., 2015; Zmora et al., 2019). Consumption of a high-fat diet (HFD) induces dysbiosis of gut microbiota, the relative abundance of Firmicutes and Proteobacteria increased, while the relative abundance of Bacteroidetes and Verrucomicrobia decreased (Walker et al., 2014b; Araujo et al., 2017; Wang et al., 2018). These changes were seen in the intestinal bacteria of both obese (Zhang et al., 2015; Chambers et al., 2019; Sergeev et al., 2020a) and NAFLD patients (Del Chierico et al., 2016; Hrncir et al., 2021), leading to metabolic dysfunction, insulin resistance, inflammation, obesity, and T2D (Sikalidis and Maykish, 2020), a major factor causing NAFLD. In contrast, consumption of a very-low-calorie ketogenic diet (VLCKD) can increase the abundance of SCFA-producing bacteria, such as Lactobacillus and Bifidobacterium spp., resulting in amelioration of adipose tissue inflammation in obesity and NAFLD (Cunha et al., 2020; Alsharairi, 2021).

Diet not only changes composition of intestinal bacteria but also is an important factor in changing intestinal metabolites such as amino acids, fatty acids, bile acids, and other metabolites (Yang et al., 2021). All the species interconnected in the gut produce an extremely diverse reservoir of metabolites from exogenous dietary components and/or endogenous compounds generated by microorganisms and the host (Agus et al., 2021).The gut microbiota can interact with the host by producing metabolites (Ye et al., 2019; Liang et al., 2021), which are small molecules (<1500 Da) representing intermediates or end-products of microbial metabolism. The beneficial or detrimental effects of specific microbiota-derived metabolites depend on the context and the host state, suggesting the primordial nature of the symbiotic microbiota in ensuring optimal health in humans. The liver and the intestine are tightly linked through the portal circulation. Consequently, intestinal metabolites primarily arriving at the liver may have pathogenic implications (Chassaing and Gewirtz, 2014; Brandl et al., 2017). It is currently believed that intestinal metabolites such as bile acids, lipids, amino acids, vitamins, and trimethylamine N-oxide are involved in regulating the occurrence and development of NAFLD. However, changes in the intestinal metabolites in NAFLD and which metabolite changes are caused by gut microbiota remain unclear.

In our experiment, 16S rDNA and Metabonomics technology were used to analyze the cecal microbiota and its metabolites in mice to study their characteristics and relationships and explore the regulation of gut microbiota and its metabolites on NAFLD development.

## Materials and Methods

### Animal Study

#### Animal Feeding and Sample Collection

Six-week-old specific pathogen-free male C57BL/6 mice (weighting 17-19 g) were purchased from Beijing Weitong Lihua Laboratory Animal Technology Co., LTD and housed at 22 ± 2°C and 50%-60% relative humidity in a specific pathogen-free facility maintained on a 12-hour light/dark cycle in the Laboratory Animal Center of Southwest Medical University. After one week of acclimatization, 20 mice were randomly divided into two groups for 12 weeks: CK group, (n=10, Standard chow diet), NAFLD group (n=10, High fat diet, HFD). The standard chow diet comprised 65.08 kcal% carbohydrates, 23.07 kcal% proteins and 11.85 kcal% fats, while the HFD diet contained 20 kcal% carbohydrates, 20 kcal% proteins and 60 kcal% fats. All experimental mice had free access to food and water. The physical activity, consumption of food and water, and defecation of experimental mice were observed daily.

At the end of the prescribed feeding period, all mice were fasted overnight and anesthetized with an intraperitoneal injection of 1% pentobarbital sodium (50 mg/kg body weight). After anesthetization, blood samples were collected from the cardiac artery. The liver samples were dissected and weighed immediately. The liver index was calculated using the following formula: liver wet weight/total body weight ×100%. After the liver was fixed with 4% paraformaldehyde, the tissue was sectioned and stained with hematoxylin-eosin (HE). The contents of the cecum were placed in liquid nitrogen and tested for the microbiome and metabolome. The experimental protocol was approved by the *Animal Ethics* Committee of Southwest Medical University (No. of *Animal Ethics* Approval: SWMU2019243).

#### Biochemical Analysis of Serum

Blood samples were acquired in the morning and centrifuged at 3500 r/min for 10 min at 4°C. Recovered supernatants were separated into 200 μl tubes and immediately frozen at -80°C. Liver function indexes such as alanine aminotransferase (ALT), aspartate aminotransferase (AST), triglyceride (TG), total cholesterol (TC), high-density lipoprotein (HDL) and low-density lipoprotein (LDL) were detected by a fully automatic veterinary biochemical analyzer.

### Microbiota Analyses

Cecal DNA was isolated using the Qiagen Gel Extraction Kit (Qiagen, Hilden, Germany). The genomic DNA was amplified using fusion primers targeting the 16S V3-V4 rRNA gene with indexing barcodes. All samples were pooled for sequencing on the Illumina HiSeq platform according to the manufacturer’s specifications. Raw pyrosequencing reads were generated from FLASH (V1.2.7, http://ccb.jhu.Edu/software/FLASH/). Quality filtering, chimera removal and *de novo* operational taxonomic units (OTUs) clustering were carried out using the Uparse (V7.0.1001, http://drive5.com/uparse/), which identifies highly accurate OTUs from amplicon sequencing data with an identity threshold of 97%. Then the OTUs were used to screen effective sequences using Mothur (http://www.mothur.org/). The representative sequences of OTUs were used to analyze alpha-diversity (Chao1, Ace, Shannon and Simpson diversity index) based on their relative abundance. A heatmap was generated according to the relative abundance of OTUs by R software (V2.15.3, http://www.R-project.org). Principal Co-ordinates analysis (PCoA) based on UniFrac distance was performed with Qiime (V1.9.1, http://qiime.org/scripts/split_libraries_fastq.html). The linear discriminant analysis (LDA) with effect size measurements (LEfSe) was used to identify indicator bacterial groups specialized within the two groups.

### Non-Targeted Metabolomics

Untargeted metabolomics were used to analyze 100 mg of cecal contents/sample. For LC-MS analysis, the samples were re-dissolved in 100 μL acetonitrile/water (1:1, v/v) solvent. Analyses were performed using a UHPLC (1290 Infinity LC, Agilent Technologies) coupled to a quadrupole time-of-flight (AB SciexTripleTOF 6600) in Shanghai Applied Protein Technology Co., Ltd. The positive and negative ionization modes of electrospray ionization (ESI) were used for mass spectrometry. The samples were separated by UHPLC and analyzed by Agilent 6550 mass spectrometer and the chromatographic and mass spectrometry conditions used are provided in the references (Luo et al., 2019).

For the data extracted using XCMS, ion peak data for which >50% of the data were missing within a group were deleted. After the data had been pre-processed by Pareto-scaling, pattern recognition was performed using SIMCA-P software (version 14.1, Umetrics, Umea, Sweden), consisting of unsupervised principal component analysis (PCA) and supervised orthogonal partial least squares discriminant analysis (OPLS-DA). The 7-fold cross-validation and response permutation testing were used to evaluate the robustness of the model. The variable importance in the projection (VIP) value of each variable in the OPLS-DA model was calculated to indicate its contribution to the classification. Metabolites with VIP value >1 were further applied to Students *t*-test at a univariate level to measure the significance of each metabolite and *p-value* less than 0.05 were considered statistically significant.

The metabolites were blasted against the online Kyoto Encyclopedia of Genes and Genomes (KEGG) database (http://geneontology.org/) to retrieve their KEGG orthologs (KOs) and were subsequently mapped to pathways in KEGG. KEGG pathway enrichment analyses were applied based on the Fisher’s exact test, considering the whole metabolites of each pathway as background dataset. Only pathways with *p-value* under a threshold of 0.05 were considered significant. The studied metabolites relative expression data was used to perform hierarchical clustering analysis. For this purpose, Cluster3.0 (http://bonsai.hgc.jp/~mdehoon/software/cluster/software.htm) and the Java Treeview software (http://jtreeview.sourceforge.net) were used.

### Bioinformatic Analysis Using Multi-Omics Integration of Metabolome and Microbiome

#### Correlation Analysis Between Metabolome and Microbiome

The Spearman statistical method was used to analyze the correlation coefficients between the significant differences and metabolites screened in the experimental samples, as well as combine the R language (V2.15.3, http://www.R-project.org) and Cytoscape software (V3.8.2, https://cytoscape.org/) to perform matrix heat mapping, hierarchical clustering, and correlation network analysis. This allowed for exploration of the relationships between microbiota and metabolites from multiple angles.

#### Model-Based Integration of Metabolite Observations and Species Abundances 2

Integration of microbiome and metabolomics data was performed using Model-based Integration of Metabolite Observations and Species Abundances 2 (MIMOSA2), freely available at http://borensteinlab.com/software_MIMOSA2.html (Mchardy et al., 2013). MIMOSA2 summarizes paired microbiome–metabolome datasets to support mechanistic interpretation and hypothesis generation. MIMOSA2 applies a method for predicting relative metabolic turnover, using a metabolic network model to translate the resulting enzymatic gene abundance estimates into community-based metabolite potential (CMP) scores. Moreover, MIMOSA2 characterizes the relative capacity of community members to produce or consume metabolites based on *a priori* metabolic information of the activity of metabolic enzymes for each species from the KEGG database. It also describes how well each metabolite can be predicted by metabolic potential and estimates how much each taxon can explain each metabolite. While correlation-based statistical analyses of metabolomic measurements are not mechanistic, this framework has the advantage of proposing mechanisms for the contributions of species to the turnover of particular metabolites. A more detailed description of this framework can be found in previously published work by the developers (Mchardy et al., 2013; Noecker et al., 2016). However, the current version of MIMOSA2 has several limitations, including the inability to capture host metabolism and it does not consider the signaling processes, transcriptional regulation, or bounds on metabolic fluxes. Nevertheless, it assigns effects for enzymes catalyzing nonreversible reactions and presumably captures major metabolic fluxes for well-characterized microbes. However, the information is lost from reversible reactions, which may hinder the prediction of metabolites in other pathways (Chorna et al., 2020).

### Statistical Analysis

Data are presented as mean ± SD. Statistical significance for body weight, fat weight, liver weight, liver index, serum indexes (TC, TG, HDL, LDL, AST, ALT), and relative abundance of bacteria were determined with Students *t*-test or Wilcoxon *Rank Sum* test. A *p-value <*0.05 was considered statistically significant. Statistical analysis was performed with SPSS software (Version 22, SPSS Inc., Chicago, USA)

## Results

### The Animal Model of NAFLD Was Successfully Established

#### Weight Gain and Fat Increase in NAFLD Mice

**Figure 1:**
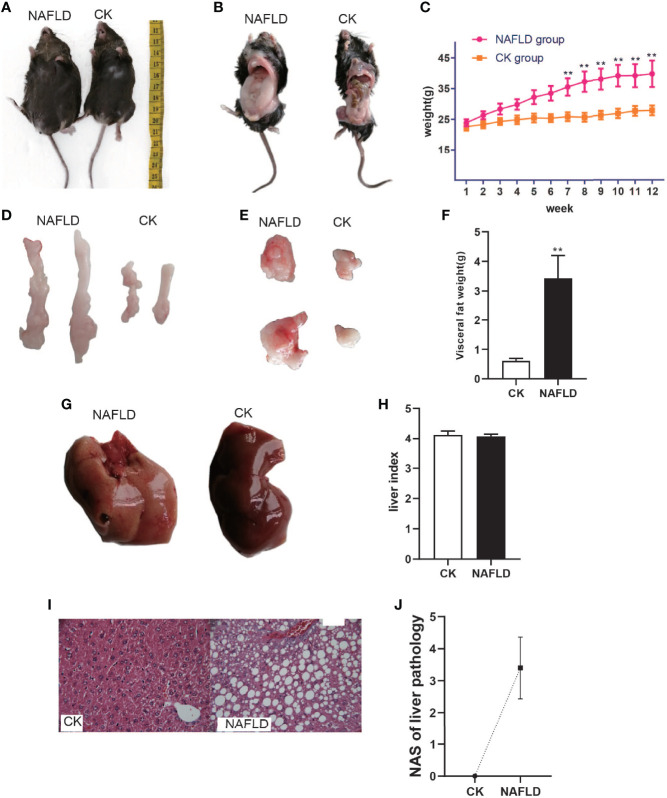
The body weight, fat mass, liver weight and Histopathology with CK and NAFLD mice, CK mice were fed Standard chow diet, NAFLD mice were fed HFD. **(A, B)** Representative pictures of CK and NAFLD mice, showing enormous discrepancy in body size. **(C)** Body weight changes in ND or HFD fed mice over 12 weeks. From week 7, the body weight of NAFLD group was significantly higher than CK group. **(D, E)** Representative pictures of perirenal fat and epididymal pad fat showing the difference of fat size between NAFLD mice and Control mice. **(F)** Tissue weight of perirenal fat and epididymal fat pads after 12 weeks of treatment. **(G)** Gross appearance of the liver. The liver of NAFLD mice was khaki yellow and that of normal mice was dark red. **(H)** There were no significant differences in liver index (% of body weight) between the groups. **(I)** Hematoxylin and eosin (H&E) staining of liver (400×), hepatocyte steatosis was obvious in NAFLD mice. **(J)** Through the NAS scoring system, the NAS score is 3.4, indicating moderate NAFLD. “**” indicated *p-value* < 0.01 (students *t*-test).

After being fed a high-fat diet for 3 months, the NAFLD mice were obviously enlarged (**Figures 1A, B**) with a markedly increased body weight (39.69 ± 4.31g, Figure 1C) and had significant differences as compared to normal mice (26.86 ± 1.08g, *P*<0.01) (**Figure 1C**). NAFLD mice had significantly more visceral fat (perirenal fat and epididymal fat) than normal mice (**Figures 1D, E**). The visceral fat weight of NAFLD mice (3.43 ± 0.77g) was significantly higher than that of the normal mice (0.61 ± 0.08, P<0.01) (**Figure 1F**).

#### Liver Steatosis in NALFD Mice

To the naked eye, the liver of normal mice appeared to be dark red while the liver of NAFLD mice was khaki yellow (**Figure 1G**). Weighing the liver and calculating the liver index (% of body weight),liver index in NAFLD mice had no significant change (**Figure 1H**). Pathological sectioning showed that normal mouse liver cells were polygonal, arranged in hepatic cords, and distributed radially around the central vein with large round nuclei in the center of the cells, uniform cytoplasm, no lipid droplets, no steatosis, or inflammatory cell infiltration. The structure of the liver lobules of NAFLD mice was disordered, with the liver cells obviously swollen and lipid droplets of different sizes were present in the cytoplasm. The fusion of the lipid droplets caused the cell nucleus to shift or even disappear and some liver cells had ballooned in varying degrees (**Figure 1I**). Through the NAS scoring system, the NAS score was 3.4 (**Figure 1J**), indicating moderate NAFLD.

**Figure 2:**
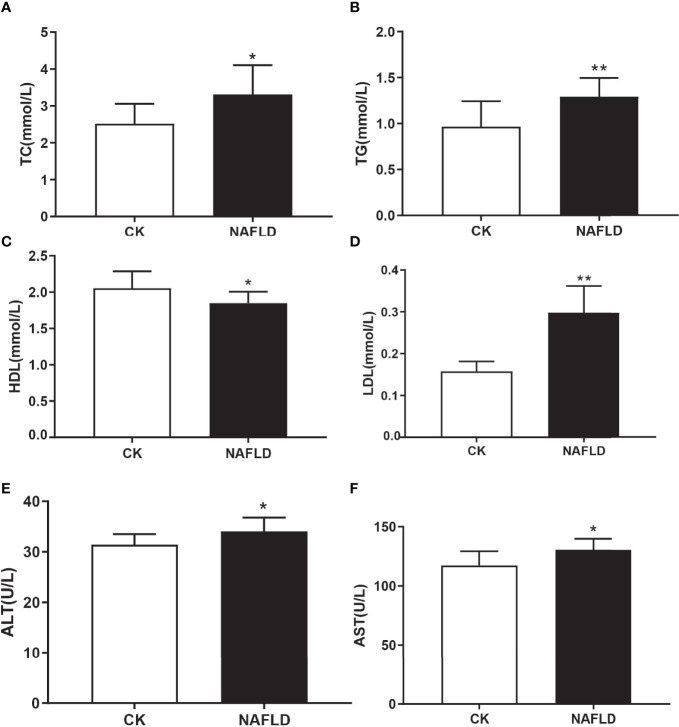
Serum lipid and liver function in Control and NAFLD mice. **(A-F)** Total cholesterol (TC), triglycerides (TG), high density lipoprotein (HDL), low density lipoprotein (LDL), aspartate aminotransferase-AST (E) and alanine aminotransferase (ALT) were significantly elevated in NAFLD mice. “*” indicated *p-value* < 0.05, “**” indicated *p-value* < 0.01 (Students *t*-test).

Serological testing found that the levels of liver injury indicators of NAFLD mice, total cholesterol (TC) (**Figure 2A**), triglycerides (TG) (**Figure 2B**), lipid indicators of low-density lipoprotein (LDL) (**Figure 2D**), alanine aminotransferase (ALT) (**Figure 2E**) and aspartate aminotransferase (AST) (**Figure 2F**), were significantly higher than the CK group. High-density lipoprotein (HDL) (**Figure 2C**) level was significantly lower than the CK group and each serum index had significant statistical significance (*P*<0.05).

### The Structure of Gut Microbiota in Mice With NAFLD Was Significantly Changed

#### The Alpha Diversity of Gut Microbiota Was Not Changed in NAFLD Mice

**Figure 3:**
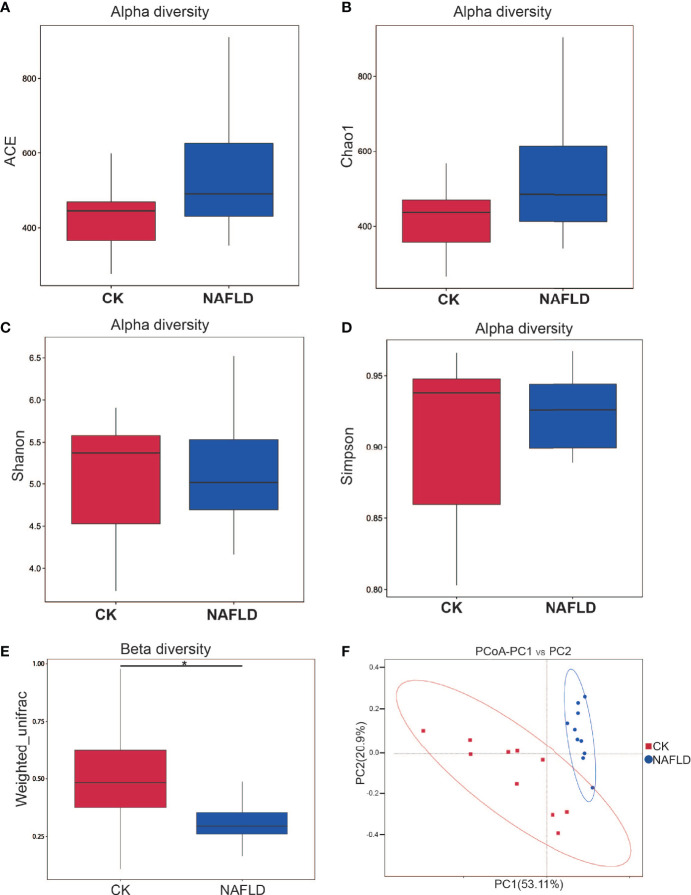
Alpha and beta diversity analysis of the bacterial community in the cecal contents of Control and NAFLD mice. **(A-D)** ACE, Chao1, Shannon, Simpson indexes had no significant difference between Control and NAFLD mice. **(E, F)** Beta diversity assessed by using PCoA of weighted UniFrac distance metrices had significant difference between Control and NAFLD mice. “*” indicated *p-value* < 0.05, (Wilcox *Rank-Sum* test).

Different metrics have been devised to measure alpha diversity with emphasis on the different aspects of the community structure: Ace, Chao1, Shannon, and Simpson indexes (**Figures 3A–D**). Ace index and Chao1 index are used to evaluate the richness of microflora, while Shannon index and Simpson index are comprehensive indexes reflecting the richness and uniformity of microflora. The results showed that all four indexes analyzed had no significant difference (*P*>0.05), indicating that HFD did not change the alpha diversity of intestinal microbiota in mice.

#### The Beta Diversity of Gut Microbiota Was Changed in NAFLD Mice

Beta diversity measuring the variations in community membership across the different groups was performed to prove the differentiation between groups using OTU abundance with weighted Unifrac metrics, weighing species abundances with phylogenetic relationships among taxa. In principal coordinate analysis (PCoA) plots of cecal microbiota, there was significant difference between normal mice and NAFLD mice (**Figures 3E, F**).

#### The Abundance of Major Bacteria Was Changed in NAFLD Mice

**Figure 4:**
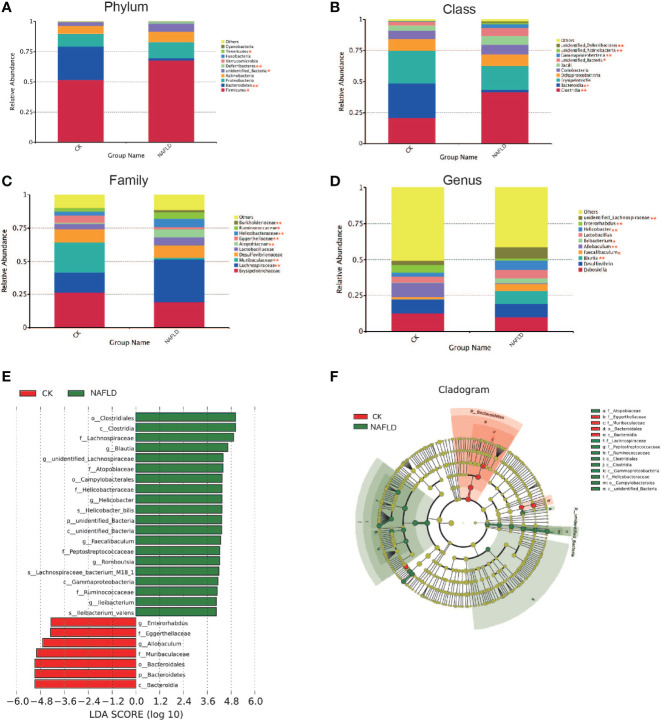
The distribution and composition of the bacterial community at each classification level. **(A-D)** Cecal bacterial profiles at the Phylum, Class, Family and Genus levels displaying relative abundance of the partial cecal microbiota. **(E)** Functional biomarkers found by linear discriminant analysis effect size (LEfSe); **(F)** Functional cladogram obtained from LEfSe. “*” indicated *p-value* < 0.05, “**” indicated *p-value* < 0.01 (Wilcox *Rank-Sum* test).

We analyzed the 10 phyla, classes, families and genera with the highest relative abundance. Intergroup comparison was done by Students *t*-test, if data were normally distributed, or otherwise by Wilcoxon *rank-sum* test. It was found that the relative abundance of Firmicutes (*P=*0.025), Unidentified_Bacteria (*P=*0.013) and Deferribacteres (*P=*0.001) increased significantly, while Bacteroidetes (*P*<0.001) and Tenericutes (*P=*0.01) decreased significantly at the phylum level (**Figure 4A**). At the class level, *Clostridia* (*P<*0.001), Unidentified_Bacteria (*P=*0.013), Gammaproteobacteria (*P=*0.007), Unidentified_Actinobacteria (*P=*0.001) and Unidentified_Deferribacteres (*P=*0.001) increased significantly, while Bacteroidia (*P<*0.001) decreased significantly (**Figure 4B**). At the family level, *Lachnospiraceae* (*P=*0.002), *Atopobiaceae* (*P<*0.001), *Helicobacteraceae* (*P=*0.004), *Burkholderiaceae* (*P=*0.005) and *Muribaculaceae* (*P<*0.001) increased significantly, while *Eggerthellaceae* (*P=*0.002) and *Ruminococcaceae* (*P=*0.038) decreased significantly (**Figure 4C**). At the genus level, *Blautia* (*P=*0.003), *Ileibacterium* (*P=*0.049), *Faecalibaculum* (*P=*0.034), *Helicobacter* (*P=*0.004) and *unidentified_Lachnospiraceae* (*P=*0.003) increased significantly, while *Allobaculum* (*P=*0.001) and *Enterorhabdus* (*P=*0.003) decreased significantly (**Figure 4D**). Through LEfSle analysis, two different phyla (Unidentifined_Bacteria and Bacteroides), four classes (Bacteroides, Clostridium, Gamma-Proteobacteria and Unidentified-Bacteria), three orders (Bacteroides, Clostridium and Campylobacter), seven families (*Lachnospiraceae*, *Helicobacteraceae*, *Eggerthellaceae*, *Muribaculaceae*, *Atopobiaceae*, *Peptostreptococcaceae* and *Ruminococcaceae*), eight genera (*Helicobacter*, *Blautia*, *Romboutsia*, *Ileibacterium*, *Faecalibaculum*, *Enterorhabdus*, *Allobaculum* and *Unidentified-Lachnospiraceae*) and three species (*Helicobacter-bilis*, *Lachnospiraceae-bacterium-M18-1* and *Ileibacterium-valens*) were found (**Figures 4E, F**).

### Intestinal Metabolite Profiles in NAFLD Mice Were Changed

To explore the mechanism by which intestinal microbiota influences the formation of NAFLD, we used an untargeted metabolome to detect cecal contents in mice. Firstly, we compared the total ion chromatograms (TIC) of 10 QC samples in positive or negative ion modes, including the retention time (RT), peak, intensity and degree of separation. Overlap of the TIC of QC samples was good, indicating that the method used was robust, with high repeatability and stability. The sample TIC showed that the peak shape was intact and that adjacent peaks were well separated from each other, indicating that the chromatographic and mass spectrometric conditions were suitable for sample identification (**Figures S1A, B**). Pearson correlation analysis was conducted on QC samples. Correlation coefficients of three QC samples in positive and negative ion mode were all greater than 0.99, indicating good correlation between QC samples (**Figures S1C, D**). QC samples fluctuated within the range of positive and negative three standard deviations in MCC, indicating that the test data were reliable (**Figures S1E, F**).

The PCA score plot showed that the interpretation rates of model (R^2^X) for normal mice and NAFLD mice under the positive and negative ion mode conditions were R^2^X=0.554 and 0.569 (**Figures S2A, B**), respectively. The two groups of samples were well separated and samples in the same group were well aggregated together (**Figures S2A, B**). The OPLS-DA supervised model was used to highlight the differences between groups. In the positive ion mode of the OPLS-DA score plot, R^2^X=0.438, R^2^Y=0.987(**Figure S2C**), Q^2 =^ 0.962, whereas in the negative ion mode, R^2^X=0.435, R^2^Y=0.992, Q^2 =^ 0.948 (**Figure S2D**). Both R^2^Y and Q^2^ values were close to 1, indicating that the model was stable and reliable. The OPLS-DA models were validated based on interpretation of variation in Y (R2Y) and forecast ability based on the model (Q2) in cross-validation and permutation tests by applying 200 iterations. The Q2 intercept values were less than 0.05, indicating that there was no overfitting and the OPLS-DA model had good predictability (**Figures S2E, F**). We used Fold Change Analysis (FC) and Students *t*-test to obtain the FC value and P value respectively to make a volcano map. In this experiment, using FC>1.5 and *P*<0.05 as the screening conditions to obtain the volcano map, 123 metabolites were screened in the positive ion mode and 78 metabolites were screened in the negative ion mode. All metabolites in the positive and negative ion mode were seen to be normally distributed. By combining the VIP values obtained from the OPLS-DA model and using VIP>1 and *P*<0.05 as the screening conditions for significantly different metabolites, 167 significantly different metabolites were obtained (**Figures S3A, B**).

**Figure 5:**
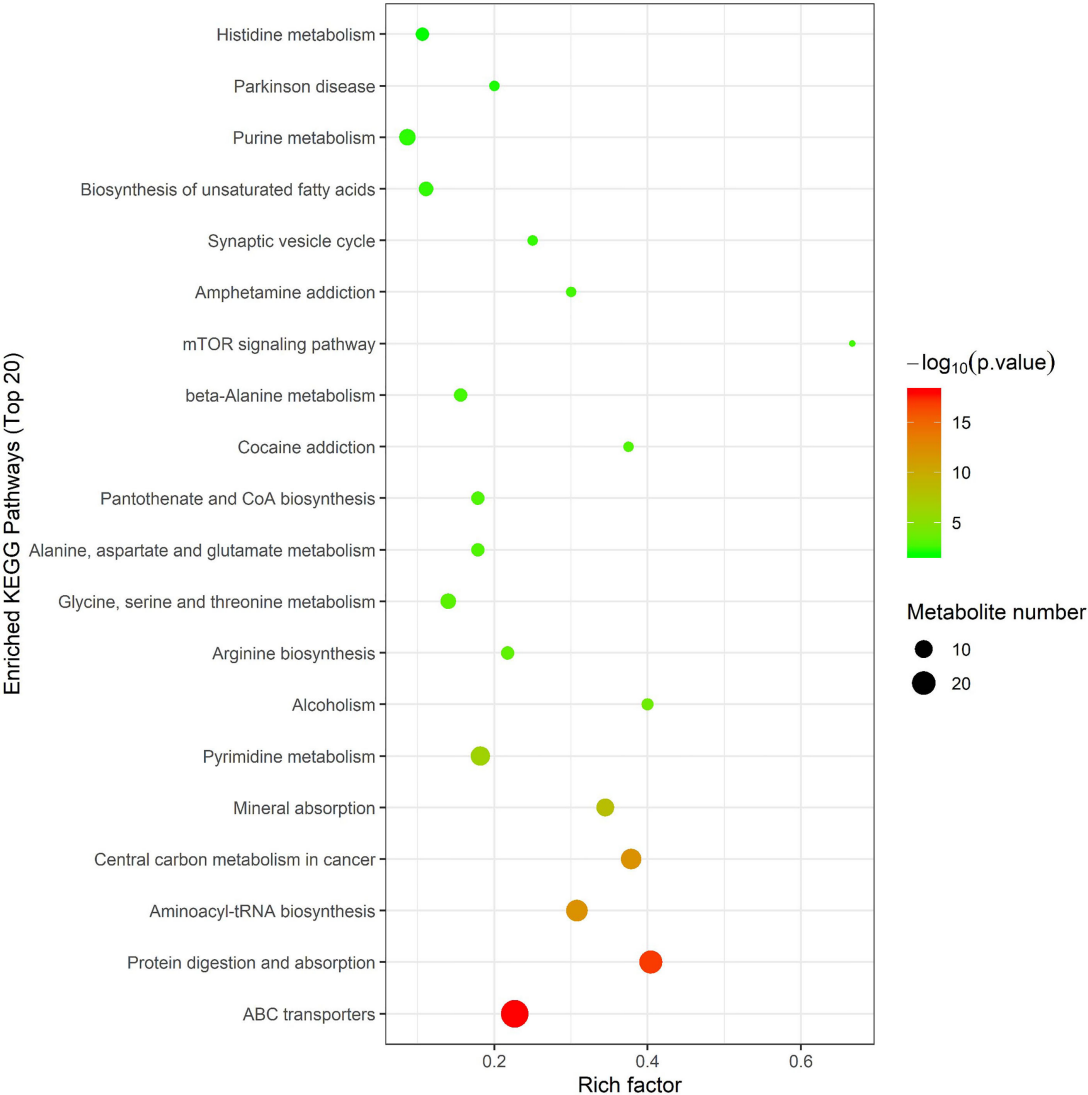
Metabolic pathway analysis using MetaboAnalyst 4.0 (http://www.metaboanalyst.ca). x-axis: Rich factor, y-axis: metabolic pathway. Circle color represents -log_10_(*p-value*) and the size of the circle represent the number of metabolites enriched in metabolic pathway.

In order to further screen the marker metabolites, the 167 significantly different metabolites selected in this experiment were analyzed by Hierarchical Clustering and KEGG metabolic pathways through the MetaboAnalyst 4.0 online analysis platform. The enrichment results of KEGG pathway (top 20) are shown in **Figure 5**, indicating that metabolites are mainly involved in the following metabolic pathways, including ABC transporter, protein digestion and absorption, aminoacyl-tRNA and arginine biosynthesis, glycine, serine and threonine metabolism, alanine, aspartic acid and glutamate metabolism, histidine metabolism, arginine and proline metabolism, unsaturated fatty acid biosynthesis, fatty acid biosynthesis, Pyrimidine metabolism, and purine metabolism.

### Some of the Metabolite Changes Are Caused by the Gut Microbiota

**Figure 6:**
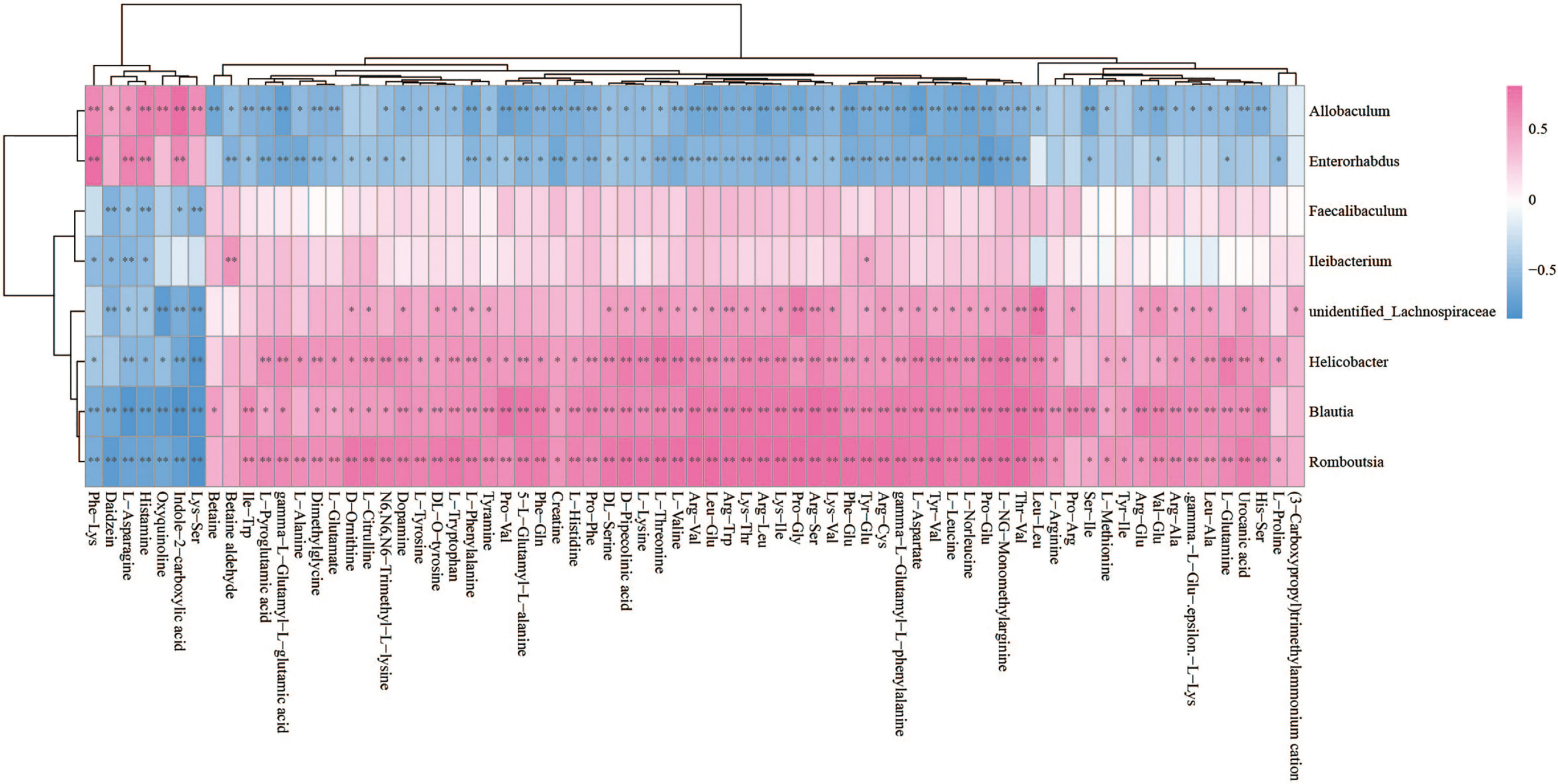
Spearman correlations between significant bacteria based on LEfSe and differential amino acids and their derivatives are presented in the form of a correlation coefficient matrix heat map. The correlation coefficient r is shown in color. r >0, positive correlation, shown in red; r < 0 represents a negative correlation, shown in blue, and the darker the color, the stronger the correlation. “*” indicated p-value < 0.05, “**” indicated p-value < 0.01.

**Figure 7:**
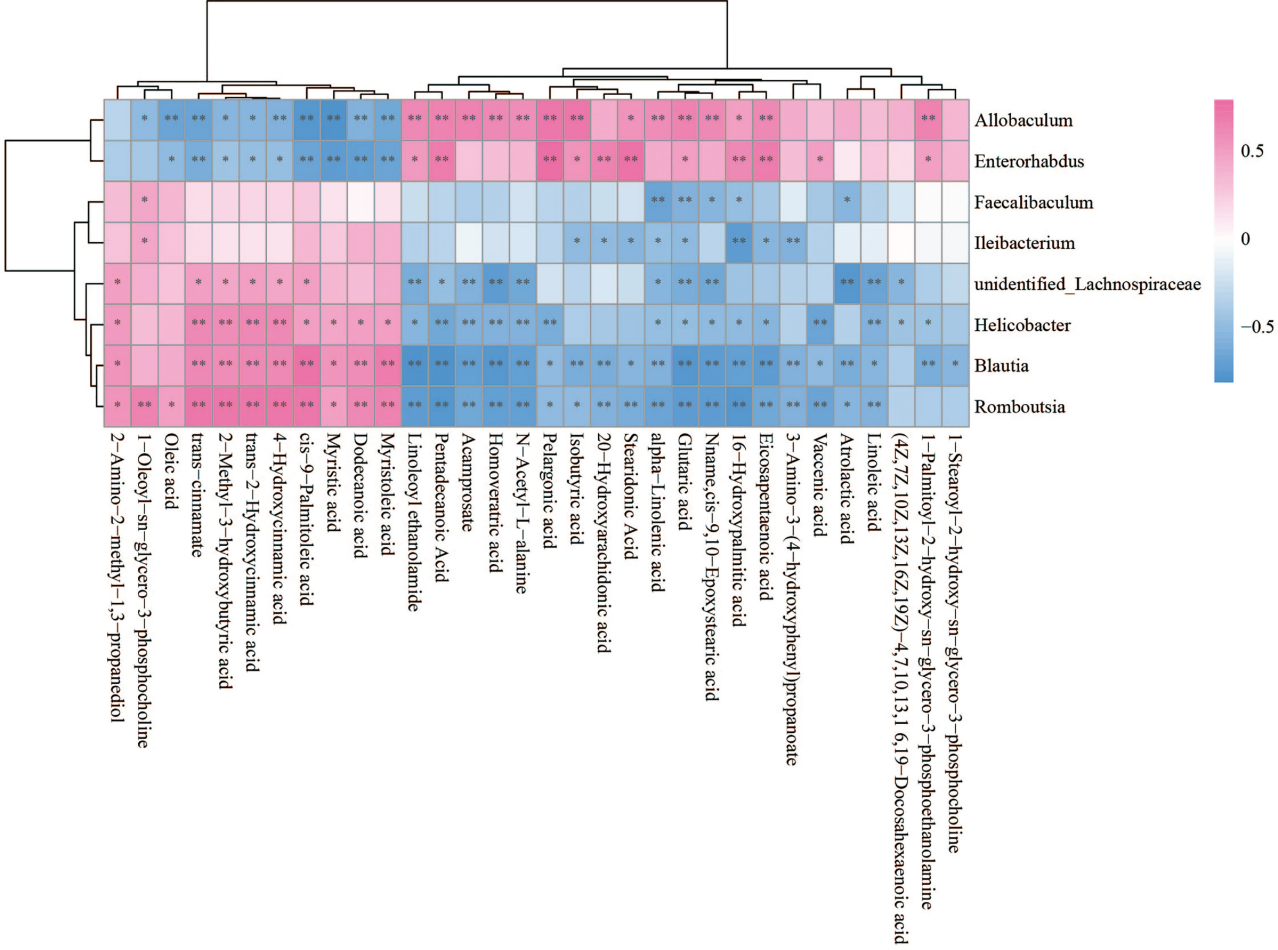
Spearman correlations between significant bacteria based on LEfSe and differential lipid are presented in the form of a correlation coefficient matrix heat map. The correlation coefficient r is shown in color. r >0, positive correlation, shown in red; r < 0 represents a negative correlation, shown in blue, and the darker the color, the stronger the correlation. “*” indicated p-value < 0.05, “**” indicated p-value < 0.01.

**Figure 8:**
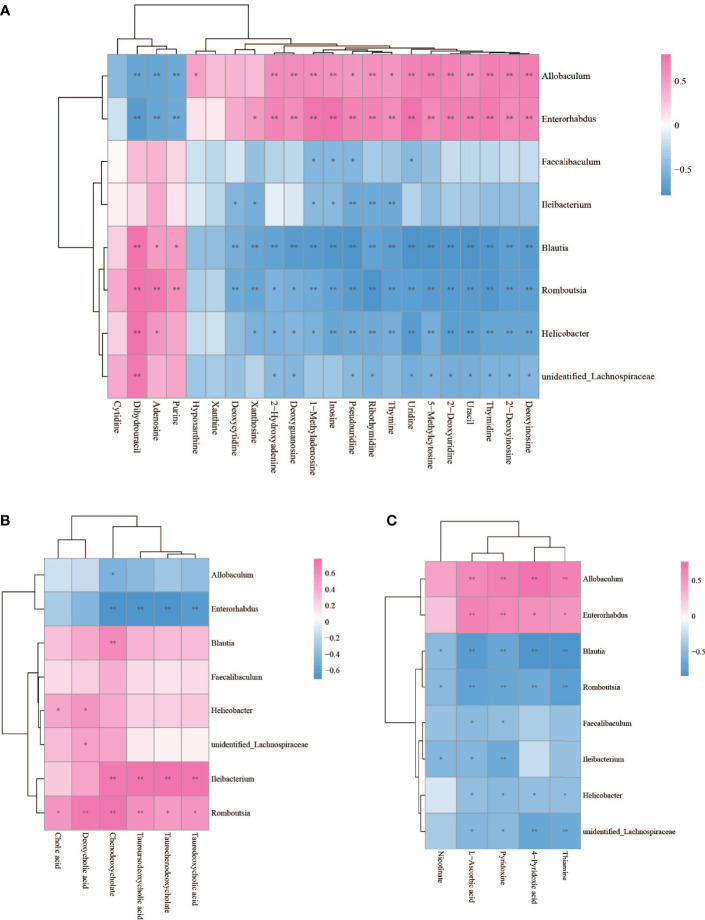
Spearman correlations between significant bacteria based on LEfSe and differential nucleotide **(A)**, bile acid **(B)**, vitamin **(C)** are presented in the form of a correlation coefficient matrix heat map. The correlation coefficient r is shown in color. r >0, positive correlation, shown in red; r < 0 represents a negative correlation, shown in blue, and the darker the color, the stronger the correlation. “*” indicated *p-value* < 0.05, “**” indicated *p-value* < 0.01.

The Spearman statistical method was used to analyze the correlation coefficients between the 8 genera with significant differences in the cecal microbiota screened by 16S rDNA technology and the 167 differential metabolites screened by metabolomics (**Figure S4**). The results showed that 8 kinds of genera are related to 70 amino acids and their derivatives (**Figure 6**), 32 lipids (**Figure 7**), 6 bile acids (**Figure 8A**), 20 nucleotides (**Figure 8B**) and vitamins (**Figure 8C**). However, whether intestinal bacteria are involved in the metabolism of these 167 metabolites is still unclear.

**Figure 9:**
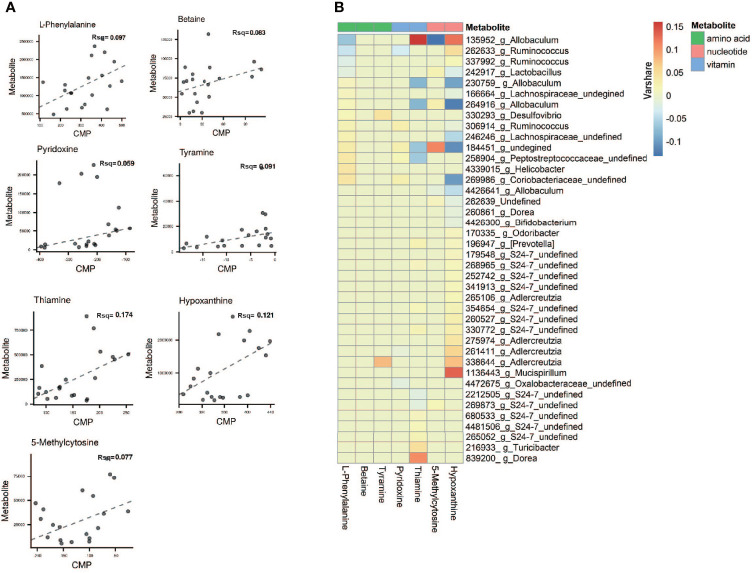
Microbial contribution to metabolite variance based on MIMOSA2. **(A)** Comprison plots showed the overall relationship between community-level metabolic potential scores (CMP) and metabolite measurements. R-square (Rsq) of the regression model used for prediction represents the sum of the contributions of all listed taxa to the metabolite variance. Contribution results are only included for metabolites with a model *p-value* less than 0.1 and positive slope of model. **(B)** Contribution heatmap showed the major taxonomic contributors with Varshare more than 0.01 to variation for each metabolite, red represents positive contribution; blue represents negative contribution; the darker the color, the greater the contribution. “Varshare” represents the fraction of the variation in each metabolite explained by the taxon in question, according to the overall community model.

To evaluate the relative ability of members of the cervicovaginal microbial community in each sample groupings to produce or utilize individual metabolites (Chorna et al., 2020), we compared the contribution of individual species to the calculated community metabolic profile (CMP) scores on the MIMOSA2 website. MIMOSA2 is an extension of MIMOSA, a novel framework for mechanistically linking microbiome ecology and metabolomic data (Noecker et al., 2016; Nalbantoglu and Sayood, 2019; Chorna et al., 2020; Gallardo et al., 2020). Well-predicted metabolites were identified by the CMP score model in CK and NAFLD groups by examining the total pool of metabolites with a positive model slope and a model *p-value*<0.1. The gut microbiota contributed to the changes in the abundance of 7 metabolites: thiamine, hypoxanthine, L-phenylalanine, tyramine, betaine, 5-Methylcytosine and pyridoxine (**Figure 9A** and **Table S2**). According to the literature, of the seven metabolites, betaine, thiamine, phenylalanine and tyramine exert a potential inhibitory effect on fatty liver. By MIMOSA2 analysis, intestinal microbiota promoted upregulation of betaine, L-phenylalanine, and tyramine and down-regulation of thiamine and pyridoxine. An unknown bacterium (Greengene ID#184451) has the ability to express betaine-aldehyde dehydrogenase (KEGG ID#K00130) and is the major bacterium responsible for the change in betaine abundance. f_*Coriobacteriaceae* (Greengene ID#269986), g_*Helicobacter* (Greengene ID#4339015), f_*Peptostreptococcaceae* (Greengene ID#258904), f_*Lachnospiraceae* (Greengene ID#246246) and unknown bacterium (Greengene ID#184451) can express chorismate mutase (KEGG ID#K14170**),** phenylalanyl-tRNA synthetase alpha chain (KEGG ID#K01889) and phenylalanyl-tRNA synthetase beta chain (KEGG ID#K01890), and are the main bacteria causing the upregulation of L-phenylalanine in NAFLD mice. Dorea (Greengene ID#839200) and g_*Turicibacter*(Greengene ID#216933), which can express Monoamine oxidase (KEGG ID#K00274), are the main bacteria causing the upregulation of tyramine. g_*Ruminococcus* (Greengene ID#306914), unknown bacterium (Greengene ID#184451) and f_*Coriobacteriaceae* (Greengene ID#269986), which can express pyridoxine kinase (KEGG ID#K00868), are the main bacteria causing the downregulation of pyrdoxine. g_*Allobaculum* (Greengene ID#135952) can express thiamine phosphate phosphatase (KEGG ID#K06949) and since its downregulation promotes thiamine downregulation, it is also one of the main reasons for L-phenylalanine upregulation (**Figure 9B** and **Table S2**).

## Discussion

In order to explore the role of gut microbiota and its metabolites in the formation of NAFLD, we induced liver steatosis by HFD. We used HFD induction rather than methionine-choline deficiency diets because HFD models better mimic fatty liver disease in humans (Arao et al., 2020). Studies also showed that while the HFD-induced NAFLD mouse model was reversible, the liver of NAFLD mice induced by methionine deficiency diet showed obvious inflammation and liver fibrosis, which was in an irreversible state. Therefore, it is recommended to use HFD induction if studying the early stages of fatty liver and methionine-choline deficient feed induction if studying the severe stages of fatty liver. Only about 20% of NAFLD in humans will enter the fibrosis stage (Matteoni et al., 1999; Sanyal, 2019). While our histopathological section also showed significant steatosis of the liver, it was still mainly steatosis without massive hepatocyte necrosis and fibrosis.

The cecal contents of mice was collected as the research focus. Alpha diversity analysis based on 16S rDNA showed no significant differences in the Chao1, Ace, Shannon, and Simpson indexes between NAFLD mice and normal mice (**Figures 3A–D**). The results indicate that the diversity and richness of gut microbiota in NAFLD mice did not change. The beta diversity analysis showed a significant change in the PCoA index, indicating that while the HFD had not changed the types of bacteria taxa present, it had changed the relative abundance of bacteria at different classification levels. Thus, the structure of gut microbiota was changed but since the changes were not severe, it was still in the reversible stage. This is also a common feature of gut microbiota after HFD induction (Walker et al., 2014; Xiao et al., 2017; Wallis et al., 2020). After returning to a normal diet, the gut microbiota returns to its original characteristics (Xiao et al., 2017). Consequently, we propose that the changes in intestinal structure are mainly caused by diet (Xiao et al., 2017).

At the phylum level, it was found that Firmicutes in the NAFLD mice increased significantly while the Bacteroides decreased significantly. This is supported by the results of other studies (Lambert et al., 2015). After a HFD, the abundance of murine Firmicutes increased while that of Bacteroides decreased (Walker et al., 2014). In the human gut, the abundance of Firmicutes in people with obesity (Sergeev et al., 2020), fatty liver (Lambert et al., 2015) or diabetes (Palacios et al., 2020) also increased and the abundance of Bacteroides decreased. Hildebrandt et al. (Hildebrandt et al., 2009) believed that after the normal diet was changed to a high fat diet, regardless of the obesity status of the mice, the number of Bacteroides in their gut decreased, while the numbers of Firmicutes and Proteobacteria increased. This shows that the changes in gut microbiota are due to diet instead of obesity or fatty liver.

LEfSe analysis highlights statistical significance and biological correlation and can identify diagnostic markers. LEfse analysis showed that the abundance of six bacteria genera increased significantly, while two other genera showed a significant decrease in the cecum of NAFLD mice. The abundance of *Helicobacter, unidentified_Lachnospiraceae, Blautia, Romboutisa, Faecalibaculum* and *Ileibacterium* significantly increased in our experiment. *Helicobacter* is considered as an intestinal pathogenic bacterium, belonging to *Campylobacteraceae* with *Helicobacter pylori*. Some studies believe that *Helicobacter* is a contributing factor in the development of NAFLD and that eradication of Helicobacter is an effective preventive or therapeutic measure (Sumida et al., 2015; Waluga et al., 2015) but this remains a controversial issue at present (Baeg et al., 2016; Kim et al., 2017). *Blautia* is also another controversial bacterium. The abundance of *Blautia* is positively correlated with obesity (Goffredo et al., 2016) and serum TG (Tang et al., 2018; Carbajo-Pescador et al., 2019), suggesting that *Blautia* may be involved in the early occurrence of obesity-related NAFLD. However, in some studies*, Blautia* has been shown to be a potentially beneficial bacterium (Li et al., 2016; Fu et al., 2018) which can ameliorate NAFLD or obesity (Zhang et al., 2019; Zhou and Fan, 2019; Zheng et al., 2020). There are few studies on *Romboutsia*, *Faecalibaculum* (Li et al., 2018; Hu et al., 2019) and *Ileibacterium* (Bai et al., 2019). In those studies, these bacteria were significantly increased in NAFLD mice, although their physiological effects on NAFLD are not clear. Results of LEfSe analysis showed that *Allobaculum* and *Enterorhabdus* significantly decreased in NAFLD mice. Other experiments also found that *Allobaculum* was also significantly decreased after HFD induction (Raza et al., 2017; Ghaffarzadegan et al., 2018). *Allobaculum* was confirmed to be an important functional bacterium (Zhang et al., 2012; Everard et al., 2014) that is negatively correlated with obesity, especially subcutaneous fat content (Hu et al., 2019). It has been reported that *Allobaculum*, as a beneficial bacterium, is associated with weight loss in mice with normal metabolism, manifested as insulin sensitivity and remission of systemic inflammation. Some researchers hypothesized that the increased abundance of *Allobaculum* can help young mice resist the development of obesity (Hildebrandt et al., 2009) and assist in improving the integrity of the intestinal barrier (Le Roy et al., 2013). *Enterorhabdus* is a gram-negative bacterium that exists only in mice and its association with obesity has been controversial (Guo et al., 2020). In conclusion, the relationship between these bacteria and NAFLD requires further study.

**Figure 10:**
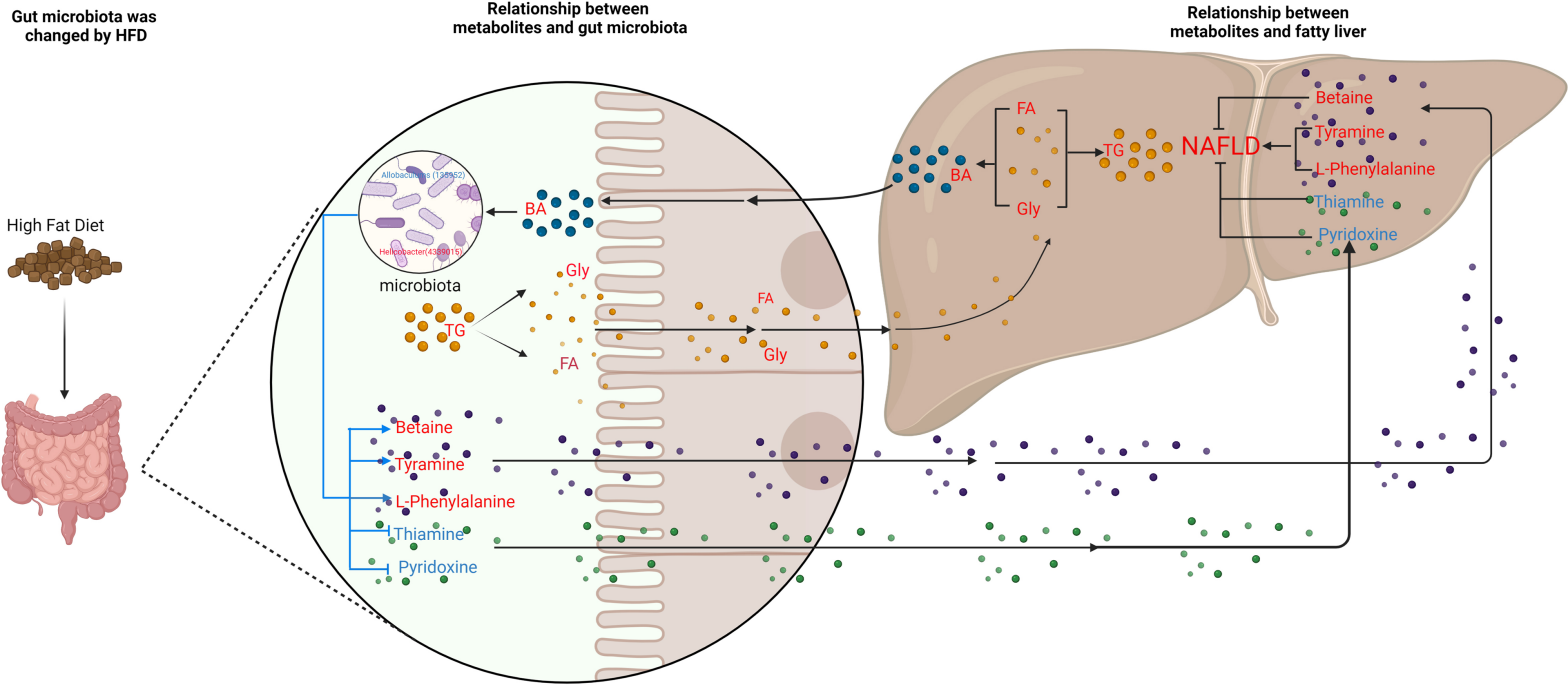
Schematic figure illustrating the relationship between gut microbiota and metabolites, and its effect on NAFLD. Red font represent up-regulated metabolites; blue font represent down-regulated metabolites. The blue line represents the pathways studied in this experiment, and the black line represents the pathways supported by the literature.

To further explore the relationship between gut microbiota and NAFLD, we performed metabolome analysis of cecal content. We successfully screened 167 differential metabolites. Through correlation analysis between differential metabolites and gut microbiota, we found that although changes in gut microbiota have a certain correlation with changes in lipid metabolites (**Figure 7**), MIMOSA2 analysis showed that the difference in microbiota did not contribute to changes in lipid metabolites (**Figure 9A** and **Table S2**). The HFD which we used contains 34% lard. Oleic acid is the largest component of lard (with a content of 31.97%-50.52%), followed by Palmitic acid (19.97%-27.75%), Stearic acid (6.37%-17.81%), LA (11.7%-23.84%) and α-LA (0.23%-2.09%) (Shestakov Iu and Piroxhnik, 1976; Jensen et al., 1999). Metabolome analysis found that Oleic acid and Palmitic acid significantly increased, while LA, α-LA, AA, Stearidonic acid, EPA and DHA were significantly reduced in NAFLD mice. Therefore, we speculated that the HFD, instead of the gut microbiota, was responsible for the decrease of ω3-PUfas and the increase of saturated fatty acids and oleic acid in the intestinal tract of NAFLD mice, thus leading to the accumulation of liver lipids (**Figure 10**).

Our experiment found that the levels of bile acids, an important metabolite in cecum of NAFLD mice, were also changed with a significant increase in six bile acids (**Table S1**). High level of TG leads to an increase in cholesterol. Since cholesterol is the precursor of bile acid production, the increase in cholesterol leads to the upregulation of bile acid. Previous studies suggest that high-fat and high-cholesterol diets alter the composition of bile acids in the gut, causing imbalances in the gut microbiota and aggravating bile acid metabolism disorders (Liu et al., 2020; Rao et al., 2021). A common link among many NASH pathogenesis pathways is the disruption of BA homeostasis. Bile acids bind to farnesoid X receptor (FXR), which is critically involved in maintaining BA, glucose, and lipid homeostasis (Chen et al., 2019). Also, dysbiosis of gut microbiota is able to modify the profile of BAs in patients with NAFLD (Aragonès et al., 2019). However, through MIMOSA2 analysis, we found that gut microbiota did not contribute to the changes in bile acids. Hence, we hypothesize that a HFD causes an increase in triglycerides and cholesterol, which in turn causes an increase in bile acids and thus, leads to changes in gut microbiota (**Figure 9A** and **Table S2**). Our experiments confirmed that the serum cholesterol content of NAFLD group was significantly higher than that of CK group and that six bile acids, CA, DCA, DTA, CDCA, LCA and TUDCA in the intestine, were significantly upregulated. Therefore, based on the correlation analysis results, we suggest that the upregulation of these six bile acids inhibited *Allobaculum* and *Enterorhabdus* but promoted the proliferation of *Helicobacter, Blautia, Unidentified-Lachnospiraceae, Romboutsia, Faecalibaculum* and *Ileibacterium* (**Figure 10**).

Metabonomics analysis found that most of the metabolites in the intestinal tract that were significantly changed were amino acids, with six amino acid metabolic pathways being enriched, including arginine biosynthesis; alanine, glutamic acid, and aspartic acid metabolism; histidine metabolism; arginine and proline metabolism; glycine, serine and threonine metabolism; valine, leucine and isoleucine biosynthesis (**Figure 5**). Previous studies have investigated how aspartate, glutamine, glutamate, arginine, alanine and L-citrulline can inhibit the formation of fatty liver. Leng et al. (Leng et al., 2014) found that aspartate can slow down LPS-induced liver injury, specifically by inhibiting the expression of pro-inflammatory factors (TNF-α and COX-2) and reversely regulating the expression of genes related to TLR4 and NOD signaling pathway. Glutamine and glutamate are the precursors of glutathione. Glutamine can improve lipid metabolism, enhance anti-inflammation, and antioxidant capacity while a variation of glutamate content can indicate metabolic damage caused by obesity (Jobgen et al., 2009a). Supplementation with glutamine in obese rats was also associated with a reduction of the proinflammatory cytokines TNF-α and interleukin-6 in serum and peripheral tissues (Greenfield et al., 2009), suggesting that glutamine may have anti-inflammatory effects. Arginine possesses antioxidative and anti*-*inflammatory capacities (Bronte and Zanovello, 2005; Gogoi et al., 2016) and participates in the ornithine cycle to promote urea formation, playing an important role in intestinal inflammation through immune responses and oxidative reactions. In addition, arginine supplementation reduces adiposity and improves glucose tolerance in obese rodents and humans (Popov et al., 2002; Fu et al., 2005; Jobgen et al., 2009b; Monti et al., 2012). Supplementation of HFD mice with alanine acutely suppresses weight gain in association with lower gene expression of fatty acid synthase in the liver and higher gene expression of adipose triglyceride lipase in the epididymal fat (Freudenberg et al., 2013). Thiago R. Araujo et al. (Araujo et al., 2016) verified that alanine and arginine supplementation effectively prevents fat deposition. Furthermore, alanine supplementation is more effective than arginine, since alanine managed to decrease the two abdominal fat stores evaluated. L-citrulline has anti-inflammatory and anti-oxidative properties and can break down fats, (Jegatheesan et al., 2015; Jegatheesan et al., 2016). According to MIMOSA2 analysis, the increase in these amino acids is not due to the action of gut microbiota, but may be caused by diet or body metabolism. Isoleucine, valine and leucine are collectively referred to branched chain amino acids (BCAAs). In our study, valine and leucine significantly increased in NAFLD mice, while isoleucine had no significant change. The metabolic dysregulation is often associated with an increase in the levels of valine and leucine (Floegel et al., 2013; Mardinoglu et al., 2014; Rosso et al., 2016; Gaggini et al., 2018). The dysregulation of BCAAs metabolism in patients with NAFLD, with BCAAs in blood and urine being higher (Hoyles et al., 2018; Zhou and Fan, 2019). High levels of BCAAs lead to obesity-related IR and glucose intolerance and serve as sensitive indicators of abnormal metabolism of insulin-related proteins (Gevi et al., 2018). Animal studies showed that BCAAs supplementation reduced obesity induced by a high-fat diet but caused significant liver damage in high-fat induced mice, which was associated with lipolysis abnormalities (Zhang et al., 2016). Therefore, BCAAs can reflect hepatic steatosis level independently of conventional metabolic risk factors and its metabolic abnormalities may to some extent precede the development of NAFLD (Kaikkonen et al., 2017). MIMOSA2 analysis showed that changes in valine and leucine were also not caused by gut microbiota. Only betaine, L-phenylalanine, and tyramine were affected by gut microbiota (**Figure 9A** and **Table S2**). Betaine, a trimethyl derivative of glycine, participates in liver metabolism as a methyl donor in the liver. It can regulate LXRα/PPARα pathway, reduce ER stress and predict cardiovascular outcomes. Therefore, it mainly plays a role in liver protection and confers antioxidant and anti-inflammatory benefits (Bertoldo et al., 2013). Phenylalanine is an aromatic amino acid along with tyrosine and tryptophan (AAAs), which degrade to produce a wide range of anti-inflammatory agents and phenolic compounds that act as toxins or neurotransmitters. AAAs have been shown to increase significantly in HFD-induced obesity and cardiovascular diseases such as type 2 diabetes (Liu et al., 2017). In our study, there was a significant increase in tyramine in NAFLD mice. Tyramine is produced by tyrosine decarboxylate under the action of certain intestinal bacteria, including *Enterococcus* and *Enterobacteriaceae*, followed by further deamination and oxidation to produce toxic substances such as phenol and paracresol, which destroy cellular structures and increase permeability (Murooka et al., 1978; Bargossi et al., 2015; Liu et al., 2017; Park et al., 2020). In addition, MIMOSA2 analysis also found that gut microbiota influenced pyridoxine and thiamine levels. Thiamine and pyridoxine inhibit the formation of fatty liver (Mayengbam et al., 2015; Kalyesubula et al., 2021). Analysis of the single OTU found that most of the strains played dual roles and it was difficult to analyze their roles in the occurrence and development of NAFLD. However, g_*Allobaculums* (GreenGene ID#135952) may inhibit the formation of NAFLD, while g_*Helicobacter* (GreenGene ID#4339015) may promote the formation of fatty liver. MIMOSA2 analysis showed that g_*Allobaculums* (GreenGene ID#135952) contributed significantly to the changes in the abundance of three metabolites. g_*Allobaculums* (GreenGene ID#135952) can produce thiamine phosphate phosphatase and promote thiamine production. Thus, thiamine was downregulated due to a decrease in the abundance of g_*Allobaculums* (GreenGene ID#135952). g_*Allobaculums* (GreenGene ID#135952) may play a role in inhibiting the formation of fatty liver. In addition, the upregulation of g_*Helicobacter* (Greengene ID#4339015) only contributed to the upregulation of L-phenylalanine. Therefore, we hypothesized that g_*Helicobacter* (Greengene ID#4339015) could promote the development of fatty liver by producing L-phenylalanine (**Figure 9B** and **Figure 10**).

## Conclusion

The gut microbiota of NAFLD mice changed significantly. The abundance of *Blautia*, *Unidentified-Lachnospiraceae*, *Romboutsia*, *Faecalibaculum*, *Ileibacterium* increased significantly in NAFLD mice, while *Allobaculum* and *Enterorhabdus* decreased significantly. A total of 167 metabolites in the intestinal tract of NAFLD mice also exhibited significant changes. Although there was a significant correlation between differential bacteria genera and differential metabolites in the cecum of NAFLD mice, MIMOSA2 analysis showed that the alterations in only seven metabolites were caused by gut microbiota, and the alterations in lipid and bile acid were not caused by gut microbiota. Gut microbiota may promote the formation of NAFLD by downregulating thiamine and pyridoxine, while upregulating L-phenylalanine and tyramine. g_*Allobaculums* (GreenGene ID#135952) may inhibit the formation of NAFLD by producing thiamine and degrading L-phenylalanine. g_*Helicobacter* (Greengene ID#4339015) promotes the formation of NAFLD by promoting L-phenylalanine production.

## Data Availability Statement

16S rDNA sequence data presented in the study are deposited in NCBI Sequence Read Archive(SRA), Accession number: PRJNA813033.

## Ethics Statement

The animal study was reviewed and approved by Ethics Committee of Laboratory Animals, Southwest Medical University.

## Author Contributions

CG and ZiZ designed and conceptualized study, analyzed the data, drafted the manuscript for intellectual content of the manuscript, carried out the statistical analysis, and interpreted the data. MH, LH, WX and JH carried out animal experiment. ZY, ZL, FZ, WL, QY, and LS analyzed the data. SC, ZhZ, JD and MZ reviewed experimental protocols and manuscripts., QY and ZR participated in the revision of the manuscript, SY conceptualized and designed the study and revised the manuscript. All authors contributed to the article and approved the submitted version.

## Conflict of Interest

The authors declare that the research was conducted in the absence of any commercial or financial relationships that could be construed as a potential conflict of interest.

## Publisher’s Note

All claims expressed in this article are solely those of the authors and do not necessarily represent those of their affiliated organizations, or those of the publisher, the editors and the reviewers. Any product that may be evaluated in this article, or claim that may be made by its manufacturer, is not guaranteed or endorsed by the publisher.
