# Corrigendum: The Microbiota and It’s Correlation With Metabolites in the Gut of Mice With Nonalcoholic Fatty Liver Disease

**DOI:** 10.3389/fcimb.2022.972118

**Published:** 2022-07-05

**Authors:** Congwei Gu, Zihan Zhou, Zehui Yu, Manli He, Lvqin He, Zhengzhong Luo, Wudian Xiao, Qian Yang, Fangfang Zhao, Weiyao Li, Liuhong Shen, Jianhong Han, Suizhong Cao, Zhicai Zuo, Junliang Deng, Qigui Yan, Zhihua Ren, Mingde Zhao, Shumin Yu

**Affiliations:** ^1^ College of Veterinary Medicine, Sichuan Agricultural University, Chengdu, China; ^2^ Laboratory Animal Centre, Southwest Medical University, Luzhou, China

**Keywords:** nonalcoholic fatty liver disease (NAFLD), mice, 16SrDNA, metabonomics, MIMOSA2

In the published article, there was an error in affiliation(s)

Due to our error, affiliation of two authors, Qigui Yan and Zhihua Ren, were incorrectly labeled as “Laboratory Animal Centre, Southwest Medical University, Luzhou, China”. The correct affiliation of the two authors is “College of Veterinary Medicine, Sichuan Agricultural University, Chengdu, China”. Instead of “Congwei Gu^1,2†^, Zihan Zhou^1†^, Zehui Yu^2†^, Manli He^2^, Lvqin He^2^, Zhengzhong Luo^1^, Wudian Xiao^2^, Qian Yang^2^, Fangfang Zhao^1^, Weiyao Li^1^, Liuhong Shen^1^, Jianhong Han^2^, Suizhong Cao^1^, Zhicai Zuo^1^, Junliang Deng^1^, Qigui Yan^2^, Zhihua Ren^2^, Mingde Zhao^2*^ and Shumin Yu^1*^”, it should be “Congwei Gu^1,2†^, Zihan Zhou^1†^, Zehui Yu^2†^, Manli He^2^, Lvqin He^2^, Zhengzhong Luo^1^, Wudian Xiao^2^, Qian Yang^2^, Fangfang Zhao^1^, Weiyao Li^1^, Liuhong Shen^1^, Jianhong Han^2^, Suizhong Cao^1^, Zhicai Zuo^1^, Junliang Deng^1^, Qigui Yan^1^, Zhihua Ren^1^, Mingde Zhao^2*^ and Shumin Yu^1*^”.

We apologize for our mistake. We stated this does not change the scientific conclusions of the article in any way. The original article has been updated.

## Publisher’s Note

All claims expressed in this article are solely those of the authors and do not necessarily represent those of their affiliated organizations, or those of the publisher, the editors and the reviewers. Any product that may be evaluated in this article, or claim that may be made by its manufacturer, is not guaranteed or endorsed by the publisher.

