# Supplementary material for: The Microbiota and It’s Correlation With Metabolites in the Gut of Mice With Nonalcoholic Fatty Liver Disease
Source: Front Cell Infect Microbiol. 2022 May 27;12:870785. doi: 10.3389/fcimb.2022.870785 (PMC9186341; doi:10.3389/fcimb.2022.870785)

**Supplementary figures**

**Fig.S1. Reliability evaluation of cecal contents metabolomics in NAFLD and Normal mice**. **(a-b)** Total ion chromatogram of QC sample; **(c-d)** Correlation map of QC samples; **(e-f)** Multivariable control chart (MCC) of QC sample. **(a, c, e)** in positive ion mode, **(b, d, f)** in negative ion mode.


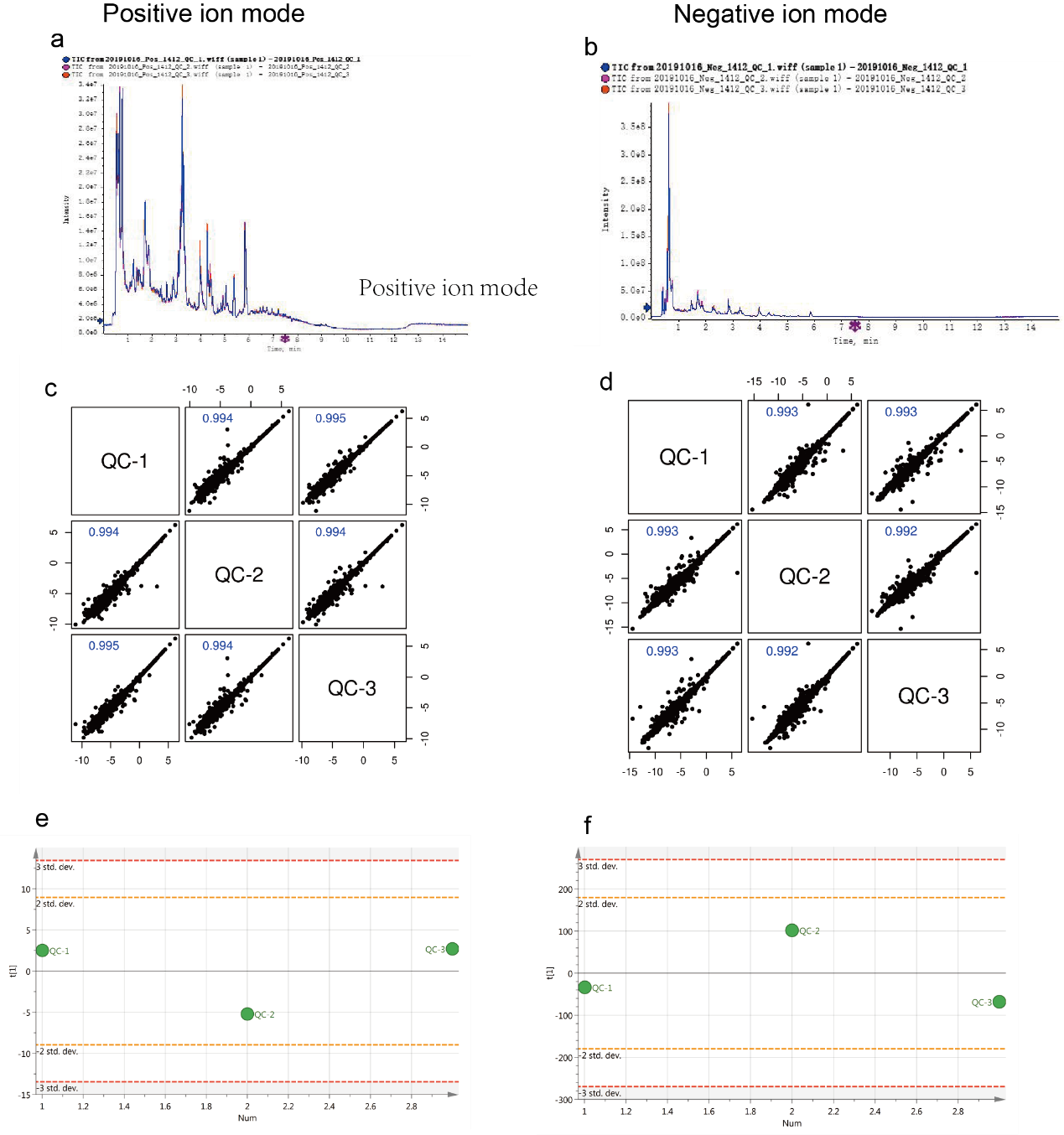


**Fig.S2.** **Non-targeted metabolomics performed with LC-MS/MS reveals microbial and host related metabolome changes** **in NAFLD and Normal mice**. **(a-b)** Principal component analysis (PCA) score plot, t[1] = first principal component, t[2] = second principal component. **(c-d)** Orthogonal partial least square discriminant analysis (OPLS-DA) of scores, t[1] = first principal component. t[2] = second principal component. **(e-f)** OPLS-DA score plot in the positive and negative ion mode. (e-f) permutation test plot of OPLS-DA. **(a, c, e)** in positive and ion mode, **(b, d, f)** in negative ion mode.


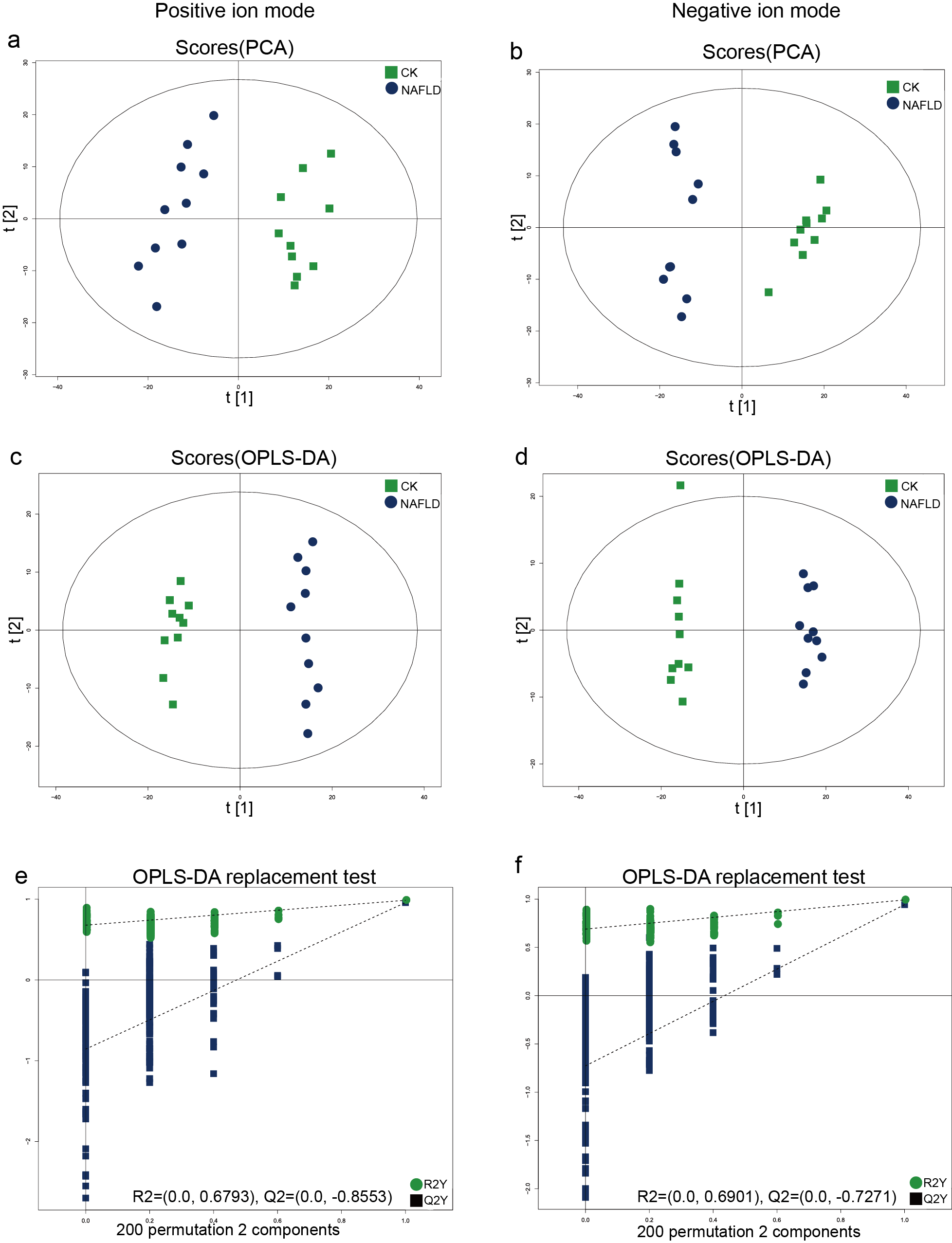


**Fig.S3. Volcano plots presenting the differential metabolites (red) between Control and NAFLD mice**. **(a)** in positive and ion mode, **(b)** in negative ion mode. Fold Change (FC) >1.5, *P*-value <0.05 (Student’s *t*-test)
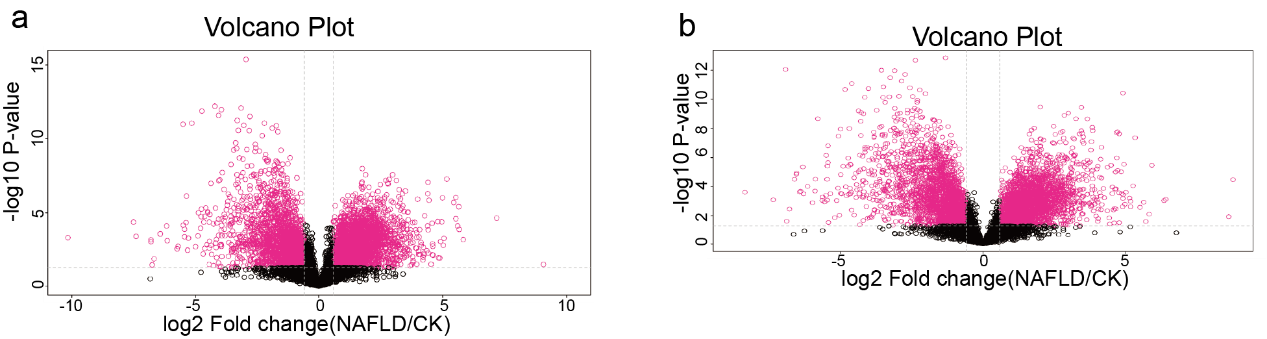


**Fig.S4. Spearman correlation between differential bactiaria based on Lefse and differential metabolites is presented in the form of a correlation coefficient matrix heat map.** r >0, positive correlation, shown in red; r < 0 represents a negative correlation, shown in blue, and the darker the color, the stronger the correlation. (*P*-value: *<0.05, **<0.01)


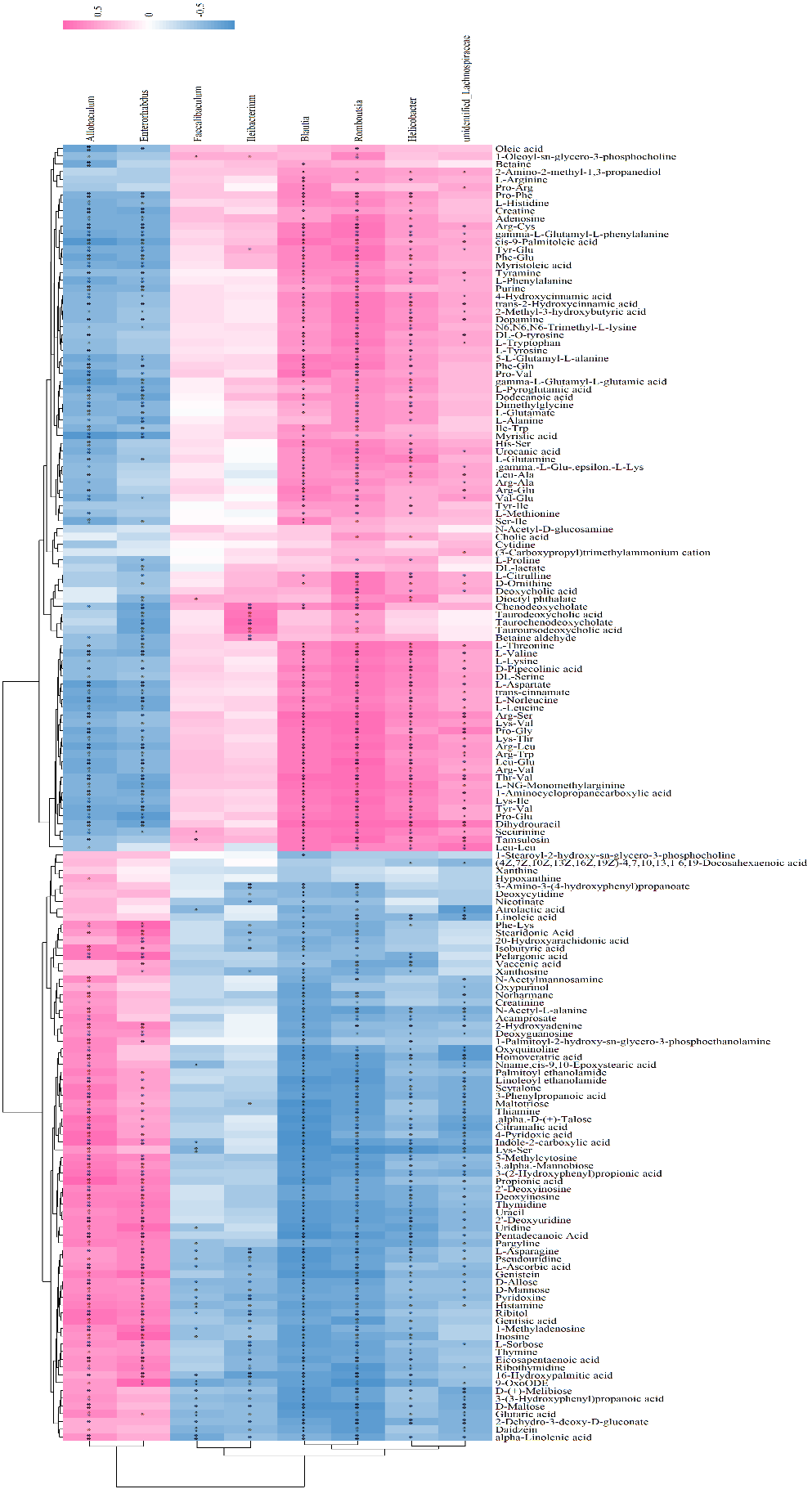

Supplement: Supplementary file 2 [file Table_2.docx]
